# Diabetic Retinopathy in the Spontaneously Diabetic Torii Rat: Pathogenetic Mechanisms and Preventive Efficacy of Inhibiting the Urokinase-Type Plasminogen Activator Receptor System

**DOI:** 10.1155/2017/2904150

**Published:** 2017-12-31

**Authors:** Maurizio Cammalleri, Massimo Dal Monte, Filippo Locri, Stefania Marsili, Liliana Lista, Mario De Rosa, Vincenzo Pavone, Dario Rusciano, Paola Bagnoli

**Affiliations:** ^1^Department of Biology, University of Pisa, Via San Zeno 31, 56127 Pisa, Italy; ^2^Department of Biology, University of Napoli Federico II, Complesso Universitario Monte Sant'Angelo, Via Cinthia, Edificio 7, 80126 Napoli, Italy; ^3^Department of Experimental Medicine, Second University of Napoli, Via Santa Maria di Costantinopoli 16, 80138 Napoli, Italy; ^4^Sooft Fidia Pharma, Contrada Molino 17, 63833 Montegiorgio, Italy

## Abstract

The spontaneously diabetic Torii (SDT) rat is of increasing preclinical interest because of its similarities to human type 2 diabetic retinopathy (DR). The system formed by urokinase-type plasminogen activator (uPA) and its receptor (uPAR) is a player in blood-retinal barrier (BRB) breakdown in DR. Here, we investigated whether in SDT rats, preventive administration of UPARANT, an inhibitor of the uPAR pathway, counteracts the retinal impairment in response to chronic hyperglycemia. Electroretinogram (ERG) monitoring was followed over time. Fluorescein-dextran microscopy, CD31 immunohistochemistry, quantitative PCR, ELISA, Evans blue perfusion, and Western blot were also used. UPARANT prevented ERG dysfunction, upregulation of vascular endothelial growth factor and fibroblast growth factor-2, BRB leakage, gliosis, and retinal cell death. The mechanisms underlying UPARANT benefits were studied comparing them with the acute streptozotocin (STZ) model in which UPARANT is known to inhibit DR signs. In SDT rats, but not in the STZ model, UPARANT downregulated the expression of uPAR and its membrane partners. In both models, UPARANT reduced the levels of transcription factors coupled to inflammation or inflammatory factors themselves. These findings may help to establish the uPAR system as putative target for the development of novel drugs that may prevent type 2 DR.

## 1. Introduction

Diabetic retinopathy (DR) is a serious complication of diabetes that accounts for the large majority of cases of adult blindness in working age population. Considering that type 1 diabetes accounts for less than 10% of all cases of diabetes [1], in most instances, DR turns out to be a disease associated to type 2 diabetes.

In type 1 diabetes, its early onset allows a precocious detection of the disease thus permitting a better control of DR, whereas, in type 2 diabetes, the late onset of DR delays its therapeutical treatments that are usually given when the disease has become vision-threating and are not always successful in restoring vision loss. Therefore, current therapeutic strategies show an unmet clinical need for therapies to prevent the occurrence and/or progression of DR in type 2 diabetes.

The streptozotocin- (STZ-) induced rodent model is an acknowledged model of type 1 diabetes in which DR is established early after diabetes onset, and its features resemble those of the early stages of DR in patients [2]. On the other hand, the literature concerning animal models of type 2 diabetes is more sparse and most developing therapies against type 2 DR have been extrapolated from the STZ model [3]. One of the best models mimicking at least in part the pathologic signs of type 2 diabetes is the spontaneously diabetic Torii (SDT) rat. The SDT rat is an inbred rat strain isolated from an outbred colony of Sprague-Dawley (SD) rats and is characterized by late diabetes onset followed by DR. Indeed, male SDT rats spontaneously develop hyperglycemia after 20 weeks of age, becoming diabetic without obesity [4]. Despite the chronic severe hyperglycemia, SDT rats survive for a long time without insulin treatment thus rendering them suitable for preventive drug efficacy studies. SDT rats are characterized by DR that becomes established at about 20 weeks after diabetes onset and is followed by severe ocular complications including upregulated expression of vascular endothelial growth factor (VEGF), structural impairment of the neuroretina and gliosis, blood-retinal barrier leakage, and reduced electroretinogram (ERG) amplitude [5].

Recently, the system formed by urokinase-type plasminogen activator (uPA) and its receptor (uPAR) has been receiving particular attention as it is likely to be a major player in BRB breakdown in the presence of DR. This system, in fact, is upregulated in response to high glucose [6–8]. Accordingly, BRB leakage can be prevented by deletion of the uPAR gene or administration of A6, a peptide that inhibits the interaction between uPA and uPAR [6, 7]. Among peptide inhibitors of the uPAR system, a tetrapeptide named UPARANT, designed to mimic the amino acid sequence through which uPAR binds its interactors in the cellular membrane, including the formyl peptide receptors (FPRs), displays resistance to enzymatic digestion, high stability in blood and plasma, and optimal effectiveness as a uPAR inhibitor [9]. UPARANT has been shown to play antiangiogenic and anti-inflammatory actions in different models of neovascular ocular pathologies [10, 11]. In the STZ rat, UPARANT has been shown to act in a therapeutic regimen by recovering the pathological signs of early DR [12], but the short-lasting duration of the disease renders this model unfit to determine possible preventive effects of the drug. Demonstrating preventive efficacy of UPARANT in models of long-lasting DR would help to establishing the uPAR system as putative target for the development of novel therapies. In the present study, we used the SDT rat as a model of late onset, long-lasting DR to investigate whether repeated systemic administration of UPARANT might prevent retinal impairment in response to persisting hyperglycemia.

## 2. Materials and Methods

### 2.1. Reagents

UPARANT was synthesized as previously described [9]. The monoclonal mouse anti-rat antibody directed against CD31 was from BD Pharmingen (San Diego, CA, USA). The AllPrep RNA/Protein Kit was purchased from Qiagen (Valencia, CA, USA). The master mix (SsoAdvanced Universal SYBR Green Supermix), the polyvinylidene difluoride membranes, and the enhanced chemiluminescence reagent were from Bio-Rad Laboratories (Hercules, CA, USA). Primers were obtained from ThermoFisher (Waltham, MA, USA). The ELISA kits were from R&D Systems (Minneapolis, MN, USA). The goat polyclonal antibody directed to claudin-1 (sc-17658), zonula occludens-1 (ZO-1; sc-8146), or FPR1 (sc-13198), the rabbit polyclonal antibodies directed to claudin-5 (sc-28670), glial fibrillary acidic protein (GFAP; sc-9065), uPAR (sc-10815), FPR2 (sc-66901), FPR3 (sc-66899), the p65 subunit of the nuclear factor kappa-light-chain-enhancer of activated B cells (NF-*κ*B p65; sc-372), NF-*κ*B p65 phosphorylated at Ser^276^ (pNF-*κ*B (Ser^276^); sc-101749), or signal transducer and activator of transcription 3 (STAT3; sc-482), the mouse monoclonal antibody directed to STAT3 phosphorylated at Tyr^705^ (pSTAT3 (Tyr^705^); sc-8059), and the mouse anti-rabbit and the rabbit anti-goat horseradish peroxidase-labeled secondary antibodies were purchased from Santa Cruz Biotechnologies (Santa Cruz, CA, USA). The rabbit monoclonal antibody directed to the active (cleaved) form of caspase-3 (number 9664) is from Cell Signaling Technology (Danvers, MA, USA). All other chemicals were purchased from Sigma-Aldrich (St. Louis, MO, USA).

### 2.2. Animals and UPARANT Treatment

**Figure 1:**
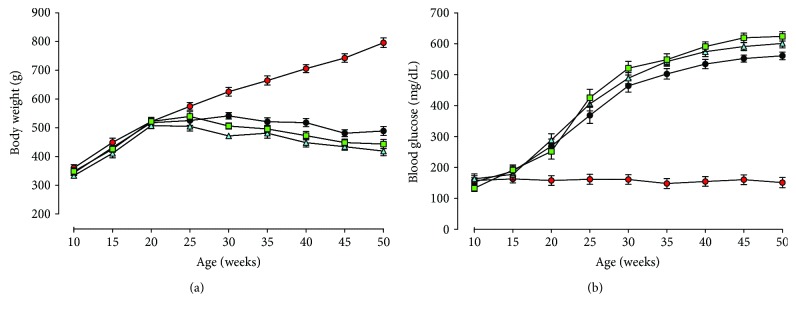
Body weight and blood glucose levels in SD and SDT rats. Mean body weight (a) and blood glucose levels (b) in SD (*n* = 9, red circles) and in SDT rats either untreated (*n* = 8, black circles), vehicle-treated (*n* = 8, light blue triangles), or UPARANT-treated (*n* = 9, green squares).

Male SDT rats (*n* = 25) were purchased from CLEA Japan Inc. (Tokyo, Japan). All animal experiments were conducted in accordance with the guidelines for care and use of experimental animals according to the European Communities Council (2010/63/UE L 27620/10/2010), the Italian law (DL: 04.03.2014, number 26), and the ARVO Statement for the Use of Animals in Ophthalmic and Vision Research. Experimental procedures were approved by the Ethical Committee in Animal Experiments of the University of Pisa. All efforts were made to minimize suffering and the numbers of animals used. In this respect, the number of animals used here was limited by the rules of the Ethical Committee due to the limited space to stock animals with long-lasting diseases in the facility. Age-matched SD rats (150–200 g) were obtained from Charles River Laboratories, Italia (Calco, Italy). Twelve of them were used as nondiabetic controls. Additional 9 SD rats were treated with a single intraperitoneal injection of 65 mg/kg STZ, dissolved in citrate buffer (pH 4.5), to induce diabetes. Blood glucose was measured by tail sampling using a OneTouch Ultra glucometer (LifeScan Inc., Milpitas, CA, USA). SDT and STZ rats were confirmed to be diabetic based on a nonfasting blood glucose values ≥ 350 mg/dL. Weight and blood glucose of nondiabetic SD or SDT rats are reported in Figures 1(a) and 1(b). No differences in weight and blood glucose were observed in SDT rats untreated, vehicle-treated, or UPARANT-treated. UPARANT was dissolved in phosphate-buffered saline (PBS) and subcutaneously administered. In SDT rats, treatments were initiated 7 weeks after diabetes onset, a time corresponding to the thirteen weeks before the occurrence of significant ERG dysfunctions. Of the SDT rats, 9 rats received UPARANT at 7 mg/kg (3 times a week), 8 rats received PBS, and 8 rats were left untreated. In STZ rats, treatments were initiated 4 weeks after diabetes onset, a time corresponding to the onset of dysfunctional ERG [12]. Of the STZ rats, 3 rats received UPARANT (8 mg/kg daily for 5 days, according to [12]), 3 rats received PBS, and 3 rats were left untreated. In all experiments, rats were anesthetized with an intraperitoneal injection of pentobarbital (30 mg/kg).

### 2.3. Electroretinographic Recording

In all SDT rats, retinal function was monitored longitudinally with scotopic full-field ERG. Before ERG testing, rats were dark adapted overnight. After anesthesia, rat pupils were dilated with 0.5% atropine and the cornea was intermittently irrigated with saline solution to prevent clouding of the ocular media. A heating pad was used to keep the body temperature at 38°C. The ERG responses were recorded from both eyes through silver/silver chloride corneal electrodes and a forehead reference electrode. A ground electrode was placed on the tail. Scotopic ERG responses, which primarily measure rod function, were triggered by flashes of different light intensities ranging from −3.4 to 1 log cd-s/m^2^ generated through a Ganzfeld stimulator (Biomedica Mangoni, Pisa, Italy). The electrodes were connected to a two-channel amplifier. Signals were amplified at 1000 gain and bandpass filtered between 0.2 and 500 Hz before being digitized at 5 kHz rate with a data acquisition device (Biomedica Mangoni). The ERG waveforms were examined primarily for amplitude information (i.e., the size of the a- and b-waves) and the data were graphed to determine any gross changes in the intensity-response function for that eye. Data were pooled and reported as mean amplitude ± SEM (in *μ*V). Intensity-response functions of the b-wave were fit to a modified Naka-Rushton equation [13].

(1) $$\begin{array}{c} V\left(I\right)=\frac{V0+\left(V\max In\right)}{\left(In+kn\right)}, \end{array}$$

where *V* is the amplitude of the b-wave (in *μ*V), *I* is the stimulus intensity (in log cd-s/m^2^), *V*0 is the nonzero baseline effect, *V*max is the saturated amplitude of the b-wave (in *μ*V), *k* is the stimulus intensity that evokes a b-wave of half-maximum amplitude (in log cd-s/m^2^), and *n*, which was constrained to unity, is a dimensionless constant controlling the slope of the function and represents the degree of heterogeneity of retinal sensitivity.

### 2.4. Fluorescein-Dextran Microscopy

Fluorescein-conjugated dextran perfusion of retinal vessels was performed in anesthetized animals using (i) 4 retinas from untreated or vehicle-treated SDT rats and (ii) 6 retinas from UPARANT-treated SDT or control SD rats. After rats were anesthetized, a median sternotomy was performed. The left ventricle was perfused with 2 mL of 25 mg/mL fluorescein-conjugated dextran in 0.15 M phosphate buffer (PB). Ten minutes after perfusion, the eyes were enucleated for fluorescein microscopy. The retinas were dissected, and flat mounts were obtained and mounted in antifade medium (Vectashield; Vector Laboratories, Burlingame, CA), vitreous side up under coverslips. Whole mounts were viewed by fluorescence microscopy (Ni-E; Nikon Europe, Amsterdam, The Netherlands). Images were acquired (DS-Fi1c camera; Nikon Europe), and an image-editing software (Adobe Photoshop CS3; Adobe System Inc., Mountain View, CA, USA) was used to create whole retina montages.

### 2.5. CD-31 Immunohistochemistry

In whole retinas from 3 different SDT or control SD rats, the retinal vasculature was visualized using antibodies directed to CD31. Dissected retinas were immersion fixed for 1.5 hours in 4% paraformaldehyde in 0.1 M PB at 4°C. They were then transferred to 25% sucrose in 0.1 M PB and stored at 4°C. The whole mounts were freeze-thawed and incubated for 72 hours at 4°C in the CD31 antibody (1 : 50 in 0.1 M PB containing 0.5% Triton X-100). They were then incubated for 48 hours at 4°C with AlexaFluor 488-conjugated secondary antibody (1 : 200 in 0.1 M PB containing 0.5% Triton X-100). Finally, the whole mounts were rinsed in 0.1 M PB, mounted on gelatin-coated glass slides, and coverslipped with a 0.1 M PB-glycerin mixture. Images were acquired with a microscope equipped with epifluorescence (Ni-E; Nikon Europe, Amsterdam, The Netherlands) through a digital camera (DS-Fi1c; Nikon Europe). An image-editing software (Adobe Photoshop CS3; Adobe System Inc., Mountain View, CA, USA) was used to create whole retina montages on which the subsequent quantification was performed.

### 2.6. Quantitative Real-Time PCR

Quantitative real-time PCR (qPCR) experiments were performed using 3 independent samples, each containing 1 retina, for each experimental condition. Total RNA and proteins were extracted using an isolation kit (AllPrep RNA/Protein Kit; Qiagen Inc.). Purified RNA was resuspended in RNase-free water and quantified using a fluorometer (Qubit; Invitrogen, Carlsbad, CA, USA). First-strand cDNA was generated from 1 *μ*g of total RNA (QuantiTect Reverse Transcription Kit; Qiagen). Real-time PCR amplification was performed with a kit (SsoAdvanced Universal SYBR Green Supermix; Bio-Rad Laboratories) on a CFX Connect Real-Time PCR Detection System and software CFX manager (Bio-Rad Laboratories). qPCR primer sets for VEGF, fibroblast growth factor-2 (FGF-2), claudin-1 and claudin-5, ZO-1, GFAP, caspase-3, uPAR, FPRs, tumor necrosis factor-*α* (TNF-*α*), interleukin- (IL-) 1*β*, and IL-6 were chosen to hybridize to unique regions of the appropriate gene sequence (see Table 1 for a complete list of assayed genes and primers). Amplification efficiency was close to 100% for each primer pair (Opticon Monitor 3 software; Bio-Rad Laboratories). Target genes were assayed concurrently with Rpl13a and Hprt, genes encoding for ribosomal protein L13A and hypoxanthine guanine phosphoribosyl transferase, respectively. Samples were compared using the relative threshold cycle (Ct method). The increase or decrease (fold change) was determined relative to control SD rats after normalization to Rpl13a and Hprt. All reactions were performed in triplicate. After statistical analysis, the data from the different experiments were plotted and averaged in the same graph.

### 2.7. Enzyme-Linked Immunosorbent Assay

Quantification of VEGF, FGF-2, TNF-*α*, IL-1*β*, and IL-6 protein levels was performed with commercially available kits using proteins purified as described above. Protein concentration was determined with a fluorometer (Qubit; Invitrogen). ELISA plates were evaluated spectrophotometrically (Microplate Reader 680 XR; Bio-Rad Laboratories) according to the manufacturers' instructions. Data were expressed as nanograms or picograms of targets per milligram of protein. All experiments were performed in duplicate.

### 2.8. Measurement of Retinal Vascular Leakage by Evans Blue Dye

Diabetes-induced leakage was evaluated by assessment of Evans blue dye extravasation using 3 retinas from 3 different control SD and untreated, vehicle-treated, or UPARANT-treated SDT rats. Anesthetized rats were injected with Evans blue dye (0.5% in PBS) into the left ventricle. For quantitative evaluation of BRB leakage, the animals were euthanized, the eyes were enucleated, and the retinas isolated and weighted. The Evans blue dye was extracted with formamide overnight at 65°C and read at 620 nm using a plate reader (Microplate Reader 680 XR; Bio-Rad Laboratories). For qualitative evaluation of outer BRB leakage, enucleated eyes were fixed in 4% paraformaldehyde. The retinas were flat mounted and examined with a fluorescence microscope (Ni-E; Nikon Europe, Amsterdam, The Netherlands), and images were acquired (DS-Fi1c camera; Nikon-Europe).

### 2.9. Western Blot Analysis

Proteins were purified as described above and supplemented with protease and phosphatase inhibitor cocktails. Aliquots of each sample containing equal amounts of protein (30 *μ*g) were subjected to SDS-PAGE. The gels were transblotted onto PVDF membranes that were blocked in 3% skim milk and incubated overnight at 4°C with primary antibodies. The goat polyclonal antibodies directed to claudin-1, ZO-1, or FPR1, or the rabbit polyclonal antibodies directed to claudin-5, GFAP, uPAR, FPR2, FPR3, NF-*κ*B p65, pNF-*κ*B p65 (Ser^276^), or STAT3, or the monoclonal antibody directed to the active form of caspase-3, or the mouse monoclonal antibody directed to pSTAT3 (Tyr^705^) were used. The antibodies were used at 1 : 200 dilutions with the exception of the antibodies directed to either GFAP (1 : 300) or the active form of caspase-3 (1 : 1000). The same membranes were reblotted with the mouse monoclonal antibody directed to *β*-actin (1 : 2500) used as the loading control. Finally, PVDF membranes were incubated for 1 h at room temperature with mouse anti-rabbit (1 : 5000), rabbit anti-goat (1 : 5000), or rabbit anti-mouse (1 : 25,000) horseradish peroxidase-labeled secondary antibodies, as appropriate, and developed with the enhanced chemiluminescence reagent. Images were acquired using the Chemidoc XRS^+^ (Bio-Rad Laboratories). The optical density (OD) of the bands was evaluated with the Image Lab 3.0 software (Bio-Rad Laboratories). The data were normalized to the level of *β*-actin, NF-*κ*B p65, or STAT3 as appropriate. All experiments were run in duplicate. After statistics, data were averaged and plotted in the same graph.

### 2.10. Statistical Analysis

Statistical significance was evaluated using one-way analysis of variance (ANOVA) followed by Newman–Keuls' multiple comparison posttest or two-way ANOVA followed by Bonferroni's multiple comparison posttest as appropriate. Despite the limited number of samples per group, a parametric analysis was performed as the data inside each of the samples were normally distributed. The results were expressed as mean ± SEM of the indicated *n* values (Prism 5.03; GraphPad software, San Diego, CA, USA). Differences with *P* < 0.05 were considered significant. After the data were collected, post hoc power analysis was conducted using the software G^∗^Power 3.0.10 in order to determine the statistical power of the results obtained in experiments with a small number of samples per group. A power value of 0.80 was considered the required minimum value to reject the null hypothesis.

## 3. Results

### 3.1. UPARANT Prevents ERG Dysfunction

**Figure 2:**
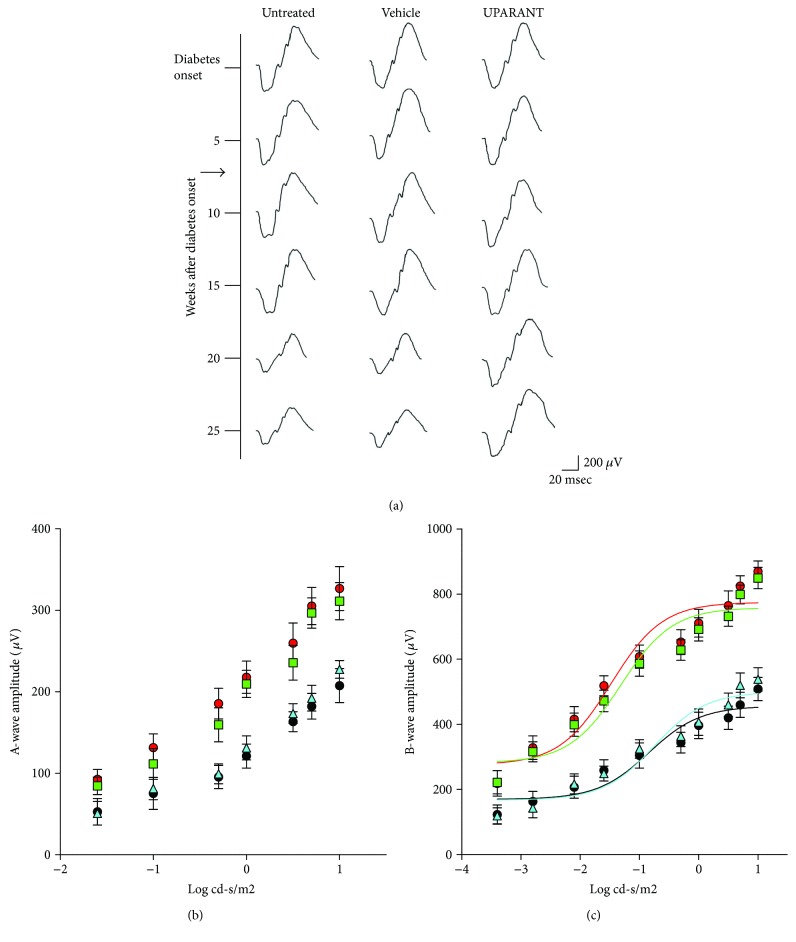
Effects of UPARANT on ERG responses. (a) Schematic representation of longitudinal ERG monitoring at different times after diabetes onset (corresponding to 20 weeks of age). The arrow indicates the beginning of the treatment (7 mg/kg UPARANT or vehicle, given subcutaneously 3 times a week for 19 weeks). The treatment was initiated at 7 weeks after diabetes onset. (b, c) Scotopic a-wave (b) and b-wave (c) amplitudes plotted as a function of increasing light intensity in control SD rats (red circles and red line) and in SDT rats, untreated (black circles and black line), vehicle-treated (light blue triangles and light blue line), or treated with subcutaneous UPARANT (green squares and green line). In respect to SD rats, both a-wave and b-wave amplitude was reduced in SDT rats, untreated or vehicle-treated. In UPARANT-treated rats, the amplitudes of the a- and b-waves did not significantly differ from those measured in SD rats (two-way ANOVA followed by Bonferroni's multiple comparison posttest). Each point represents the mean ± SEM of data from 8 (untreated and vehicle-treated SDT) or 9 (SD controls and UPARANT-treated SDT) rats.

In a longitudinal study, ERG was repeatedly recorded at different times in each SDT rat from the diabetes onset until the twenty-fifth week, before the development of severe lens opacity which might have prevented ERG evaluation. The representative recordings in Figure 2(a) show mixed a- and b-waves recorded at light intensities of 1 log cd-s/m^2^ in untreated, vehicle-treated, or UPARANT-treated rats. It can be noticed that starting from the twentieth week after diabetes onset, both in untreated and in vehicle-treated SDT rats, the ERG amplitude was decreased, in line with previous studies [14], while in UPARANT-treated SDT rats, the ERG amplitude remained unaltered over time. The diagrams in Figures 2(b) and 2(c) show amplitude reduction of both the a-wave (at light intensities ranging from −0.3 to 1 log cd-s/m^2^; *P* < 0.001) and the b-wave (at light intensities ranging from −2.8 to 1 log cd-s/m^2^; *P* < 0.001) in SDT rats, untreated, or vehicle-treated, in respect to SD control animals. In UPARANT-treated rats, the amplitudes of the a- and b-waves did not significantly differ from those measured in SD rats. Intensity-response functions of the b-wave as evaluated by a fitted Naka-Rushton equation demonstrated that the b-wave amplitude (*V*max) and the retinal sensitivity (*k*) in untreated or vehicle-treated SDT rats were significantly lower than in SD rats, whereas UPARANT treatment prevented the reduction of *V*max and *k* (Table 2).

### 3.2. UPARANT Prevents Retinal Leakage

**Figure 3:**
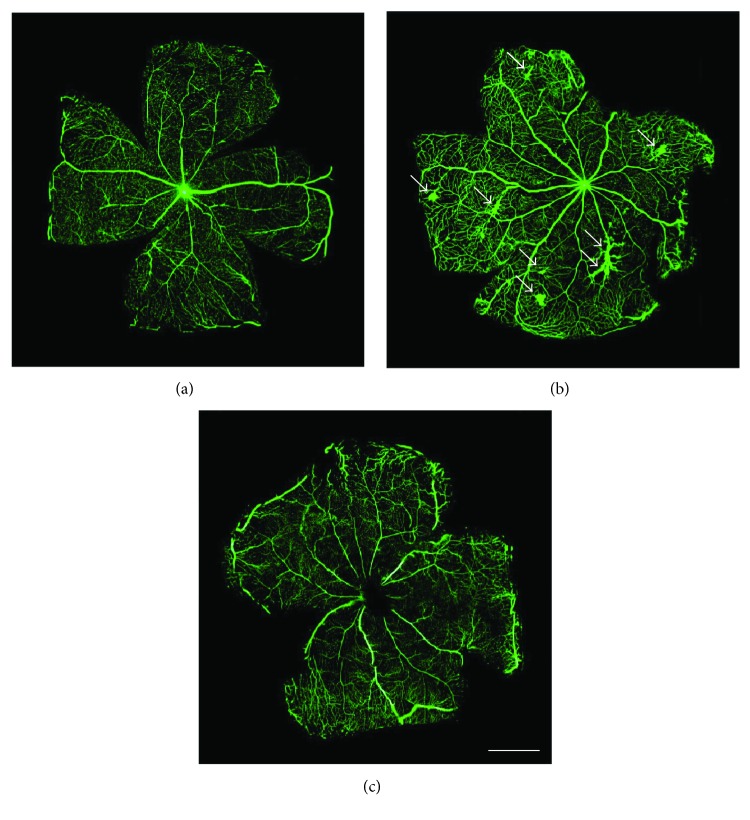
Effects of UPARANT on fluorescein leakage. (a–c) Fluorescein dextran microscopy performed soon after intraventricular injection of fluorescein solution in control SD (a) and SDT rats either vehicle-treated (b) or UPARANT-treated (c). Arrows in (b) point to hyperfluorescent areas in the retina of untreated SDT rats. In comparison with SD rats, untreated SDT rats showed abnormal retinal vasodilatation together with severe fluorescein leakage while the leakage is prevented by UPARANT administration. Scale bar: 1 mm. Fluorescein dextran microscopy was performed on 4 retinas (from untreated and vehicle-treated SDT rats) and 6 retinas (from SD control rats and UPARANT-treated SDT rats). The retinas originated from 2 or 3 different rats, respectively.

**Figure 4:**
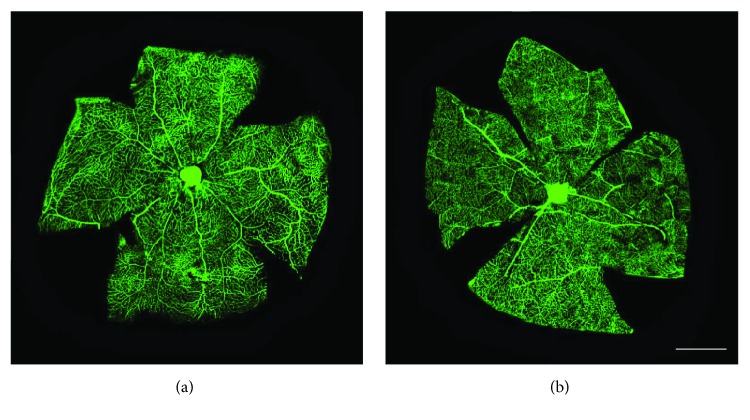
Retinal vascular phenotype. (a, b) Representive images of flat-mounted retinas from SD control rats (a) and untreated SDT rats (b) immunolabeled with a rat monoclonal antibody directed to CD31. The superficial vascular plexus is shown. No altered pattern of retinal vasculature in the superficial vascular plexus was observed in untreated SDT rats. CD31 immunohistochemistry was performed on 3 retinas from 3 different rats for each experimental condition. Scale bar: 1 mm.

**Figure 5:**
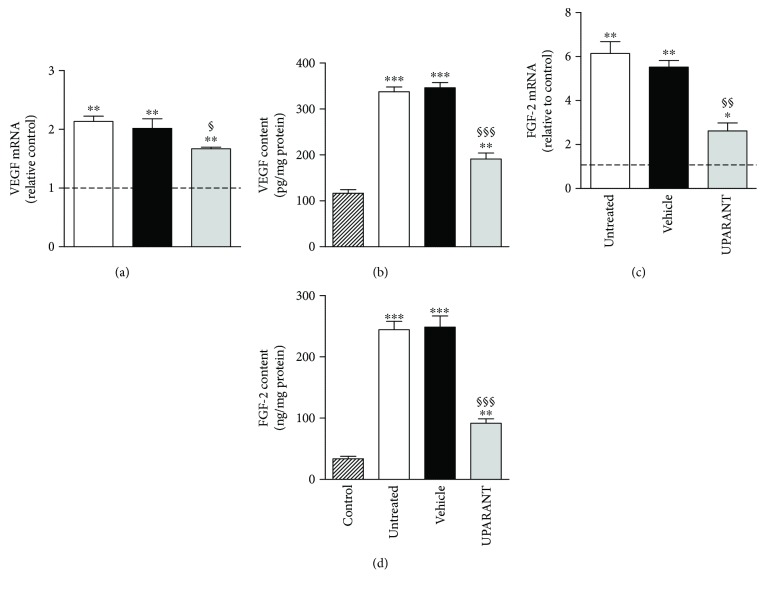
Effects of UPARANT on VEGF and FGF-2. (a, c) Transcript levels of VEGF (a) and FGF-2 (c) were evaluated by qPCR. Data were analyzed by the formula 2^−∆∆CT^ using Rpl13a and Hprt as internal standards. (b, d) Protein levels of VEGF (b) and FGF-2 (d) were evaluated by ELISA. In untreated or vehicle-treated SDT rats, levels of VEGF and FGF-2 were increased with respect to SD rats, while UPARANT treatment reduced this increase. ^∗^*P* < 0.05, ^∗∗^*P* < 0.01, and ^∗∗∗^*P* < 0.001 versus control; ^§^*P* < 0.05, ^§§^*P* < 0.01, and ^§§§^*P* < 0.001 versus vehicle (one-way ANOVA followed by Newman–Keuls' multiple comparison posttest; power values: 0.98 (a) and 0.99 (b–d)). Each column represents the mean ± SEM of data from 3 independent samples, each containing 1 retina.

**Figure 6:**
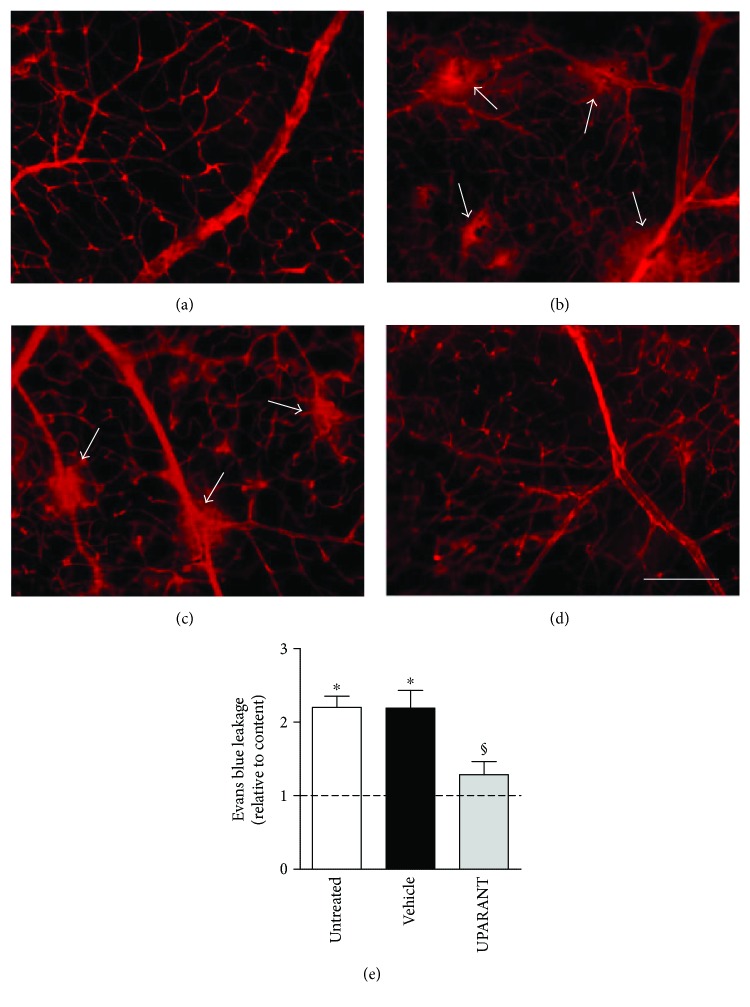
Effects of UPARANT on Evans blue leakage. (a–d) Blood-retinal vascular leakage as qualitatively evaluated with the Evans blue method in control SD (a) and SDT rats untreated (b), vehicle-treated (c), or UPARANT-treated (d). Arrows in (c) and (d) point to vascular leakage. Scale bar: 200 *μ*m. (e) Diabetes-induced leakage as evaluated by the quantitative assessment of Evans blue dye extravasation. In untreated or vehicle-treated SDT rats, Evans blue dye leakage was increased with respect to SD rats, while UPARANT treatment prevented this increase. ^∗^*P* < 0.001 versus control; ^§^*P* < 0.001 versus vehicle (one-way ANOVA followed by Newman–Keuls' multiple comparison posttest; power value: 0.84). Each column represents the mean ± SEM of data from 3 retinas from 3 different rats for each experimental condition. Three retinas from 3 different rats were used for each experimental condition.

**Figure 7:**
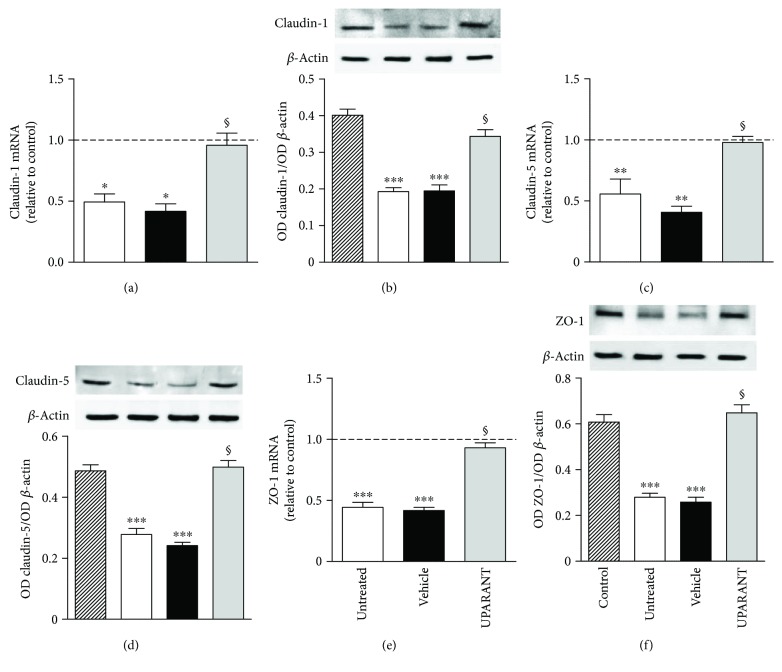
Effects of UPARANT on levels of BRB markers. (a, c, e) Transcript levels of the BRB markers claudin-1 (a), claudin-5 (c), and ZO-1 (e) were evaluated by qPCR. Data were analyzed by the formula 2^−∆∆CT^ using Rpl13a and Hprt as internal standards. (b, d, f) Protein levels of claudin-1 (b), claudin-5 (d), and ZO-1 (f) were evaluated by Western blot and densitometric analysis using *β*-actin as the loading control. In untreated or vehicle-treated SDT rats, levels of BRB markers were decreased with respect to SD rats, while UPARANT treatment prevented this decrease. ^∗^*P* < 0.05, ^∗∗^*P* < 0.01, and ^∗∗∗^*P* < 0.001 versus control; ^§^*P* < 0.001 versus vehicle (one-way ANOVA followed by Newman–Keuls' multiple comparison posttest; power values: 0.85 (a), 0.99 (b), 0.85 (c), and 0.99 (d–f)). Each column represents the mean ± SEM of data from 3 independent samples, each containing 1 retina.

Twenty-five weeks after diabetes onset, perfusion with fluorescein demonstrated that, in comparison with SD rats (Figure 3(a)), untreated SDT rats showed abnormal retinal vasodilatation together with severe fluorescein leakage (Figure 3(b)) while the leakage is prevented by UPARANT administration (Figure 3(c)). Defects comparable to those in untreated SDT rats were observed in vehicle-treated SDT rats (not shown). The retinal microvascular abnormalities evidenced by fluorescein microscopy were not accompanied by an altered pattern of retinal vasculature in the superficial vascular plexus (Figures 4(a) and 4(b)). Analysis of transcripts and proteins demonstrated upregulated levels of both VEGF and FGF2 that were prevented by UPARANT treatment (Figures 5(a)–5(d)). Fluorescein leakage was confirmed by the extravasation of Evans blue, a dye that binds to plasma proteins, and by dysregulated levels of transcripts of BRB markers. As compared to SD controls (Figure 6(a)), untreated (Figure 6(b)) or vehicle-treated (Figure 6(c)) SDT rats showed a clear extravasation of Evans blue that, on the contrary, was not observed in UPARANT-treated rats (Figure 6(d)). The quantitative analysis (Figure 6(e)) showed that in untreated or vehicle-treated SDT rats, Evans blue dye leakage was increased by approximately 2.2-fold with respect to SD rats (*P* < 0.001), while UPARANT treatment prevented this increase. Levels of transcripts and proteins of BRB markers including the transmembrane components of the interendothelial tight junctions claudin-1, claudin-5, and ZO-1 were concomitantly decreased by about 2-fold (*P* < 0.001) at the transcript (Figures 7(a), 7(c), and 7(e)) and the protein (Figures 7(b), 7(d), and 7(f)) level. UPARANT administration prevented the dysregulation of BRB markers with transcript and protein levels that did not differ significantly from those measured in SD rats.

### 3.3. UPARANT Prevents Retinal Damage

**Figure 8:**
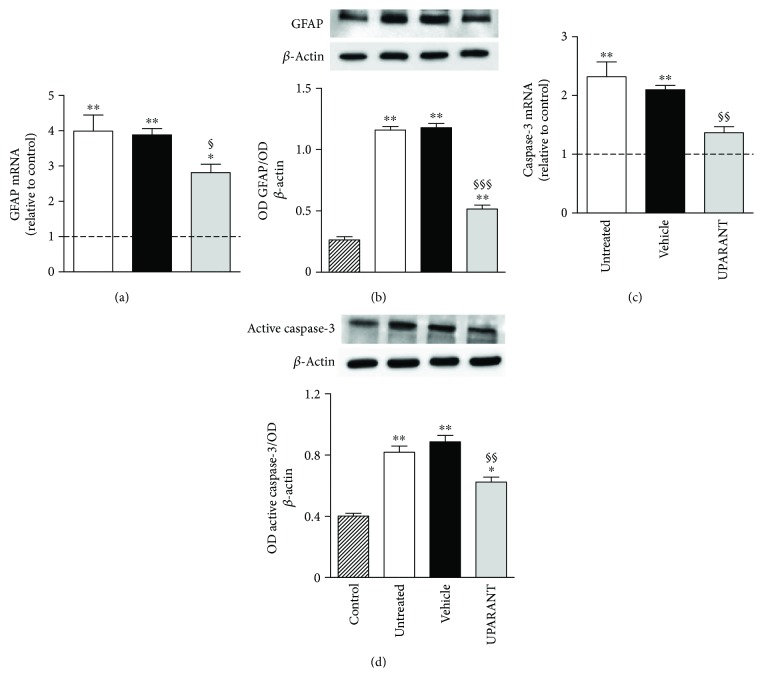
Effects of UPARANT on gliosis and retinal cell death. (a, c) Transcript levels of GFAP (a) and caspase-3 (c) were evaluated by qPCR. Data were analyzed by the formula 2^−∆∆CT^ using Rpl13a and Hprt as internal standards. (b, d) Protein levels of GFAP (b) and active caspase-3 (d) were evaluated by Western blot and densitometric analysis using *β*-actin as the loading control. In untreated or vehicle-treated SDT rats, levels of GFAP and caspase-3 were increased with respect to SD rats, while UPARANT treatment reduced this increase. ^∗^*P* < 0.01 and ^∗∗^*P* < 0.001 versus control; ^§^*P* < 0.05, ^§§^*P* < 0.01, and ^§§§^*P* < 0.001 versus vehicle (one-way ANOVA followed by Newman–Keuls' multiple comparison posttest; power values: 0.94 (a), 0.99 (b), 0.87 (c), and 0.99 (d)). Each column represents the mean ± SEM of data from 3 independent samples, each containing 1 retina.

Twenty-five weeks after diabetes onset, qPCR and Western blot demonstrated that, in comparison with SD rats, untreated SDT rats showed increased GFAP expression (Figures 8(a) and 8(b)) presumably coupled to gliotic Müller cells together with increased retinal levels of caspase-3 (Figures 8(c) and 8(d)), a reliable marker of apoptosis-induced retinal neurodegeneration [15]. Upregulated levels of both GFAP and caspase-3 were prevented by UPARANT treatment.

### 3.4. UPARANT Prevents the Upregulation of the UPAR/FPR System and Its Downstream Effectors

**Figure 9:**
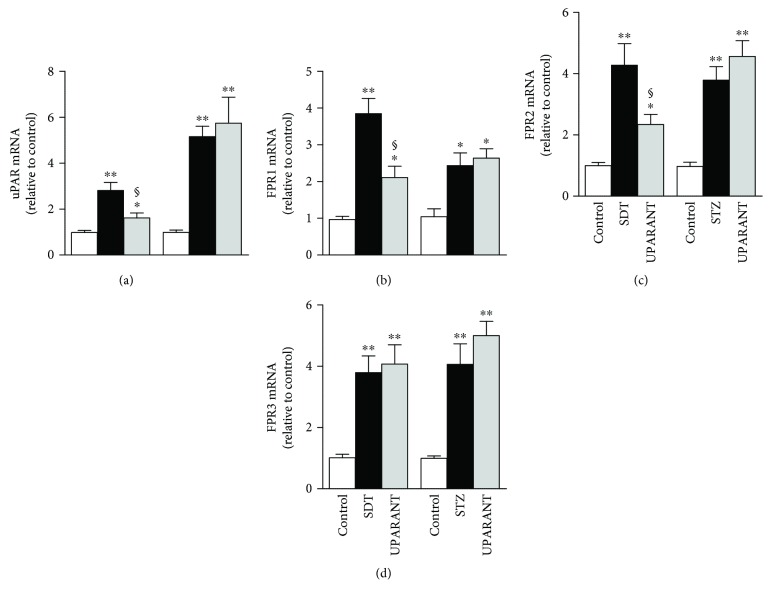
Effects of UPARANT on upregulated levels of uPAR and FPRs. (a–d) Transcript levels of uPAR (a), FPR1 (b), FPR2 (c), and FPR3 (d) were evaluated by qPCR in control SD and in SDT or STZ rats either untreated or treated with UPARANT. Data were analyzed by the formula 2^−ΔΔCT^ using Rpl13a and Hprt as internal standards. Hyperglycemia enhanced retinal levels of uPAR/FPR transcripts in both SDT and STZ rats. In SDT rats, UPARANT reduced the upregulation of uPAR, FPR1, and FPR2 without affecting FPR3. In STZ rats, no effects of UPARANT on transcript levels of uPAR or FPRs could be observed. ^∗^*P* < 0.01 and ^∗∗^*P* < 0.001 versus control SD rats; ^§^*P* < 0.01 versus untreated SDT rats (one-way ANOVA followed by Newman–Keuls' multiple comparison posttest; power values: 0.86 (a), 0.93 (b), 0.91 (c), and 0.96 (d)). Each column represents the mean ± SEM of data from 3 independent samples, each containing 1 retina.

**Figure 10:**
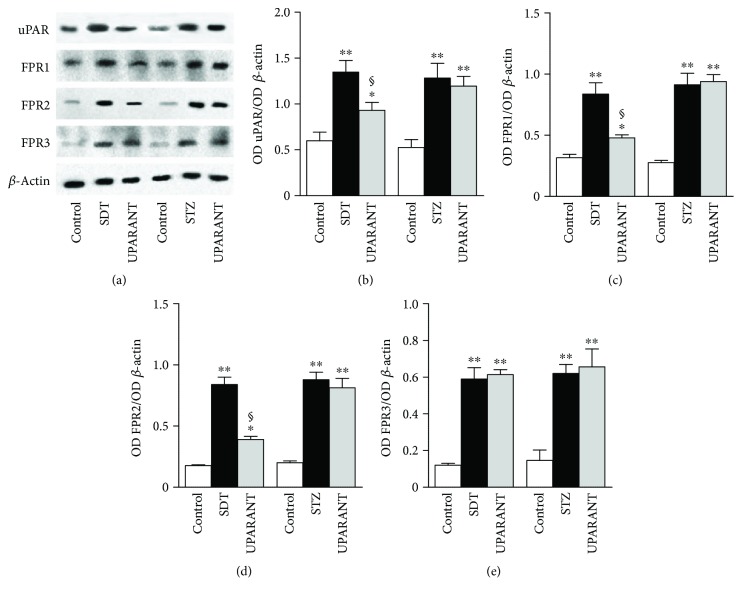
Effects of UPARANT on upregulated levels of uPAR and FPRs. (a) Representative blots from SD controls and untreated or UPARANT-treated SDT and STZ rats. (b–e) Protein levels of uPAR (b), FPR1 (c), FPR2 (d), and FPR3 (e) were evaluated by the densitometric analysis of the blots depicted in (a) using *β*-actin as the loading control. Hyperglycemia enhanced retinal levels of uPAR/FPR proteins in both SDT and STZ rats. In SDT rats, UPARANT reduced the upregulation of uPAR, FPR1, and FPR2 without affecting FPR3. In STZ rats, no effects of UPARANT on protein levels of uPAR or FPRs could be observed. ^∗^*P* < 0.01 and ^∗∗^*P* < 0.001 versus control SD rats; ^§^*P* < 0.01 versus untreated SDT rats (one-way ANOVA followed by Newman–Keuls' multiple comparison posttest; power values: 0.87 (b), 0.99 (c, d), and 0.98 (e)). Each column represents the mean ± SEM of data from 3 independent samples, each containing 1 retina.

Whether UPARANT acts through mechanisms adjusting the expression level of the respective targets and/or a regulation of intracellular effectors induced by FPR activation was determined in both SDT rats and STZ rats, which were used for comparison. We first investigated whether high glucose affects the uPAR pathway and whether the preventive action of UPARANT depends on its effects on the uPAR/FPR system. As illustrated in Figures 9(a)–9(d), hyperglycemia enhanced retinal levels of uPAR/FPR transcripts in both SDT and STZ rats. In SDT rats, uPAR transcripts were increased by about 2.8-fold (*P* < 0.001), while FPR1, FPR2, and FPR3 transcripts were increased by about 3.9-fold, 4.3-fold, and 3.8-fold (*P* < 0.001), respectively. In STZ rats, uPAR and FPR transcripts were increased by about 5.2-fold (*P* < 0.001), 2.5-fold (*P* < 0.01), 3.8-fold (*P* < 0.001), and 4.1-fold (*P* < 0.001), respectively. A similar increase was also found in vehicle-treated SDT and STZ rats (not shown). In SDT rats, UPARANT reduced the upregulation of uPAR, FPR1, and FPR2 by 1.7-fold, 1.8-fold, and 1.9-fold (*P* < 0.01) without affecting FPR3. In STZ rats, no effects of UPARANT on transcript levels of uPAR or FPRs could be observed. Transcript increase in both SDT and STZ rats was confirmed at the protein level. Representative blots from SD controls and untreated or UPARANT-treated SDT and STZ rats are depicted in Figure 10(a). The densitometric analysis (Figures 10(b)–10(e)) showed that uPAR, FPR1, FPR2, and FPR3 were increased by about 2.2-fold, 2.7-fold, 4.8-fold, and 4.9-fold (*P* < 0.001), respectively, in SDT rats, and by about 2.5-fold, 3.3-fold, 4.4-fold, and 4.1-fold (*P* < 0.001), respectively, in STZ rats. A similar increase was also found in vehicle-treated SDT and STZ rats (not shown).

**Figure 11:**
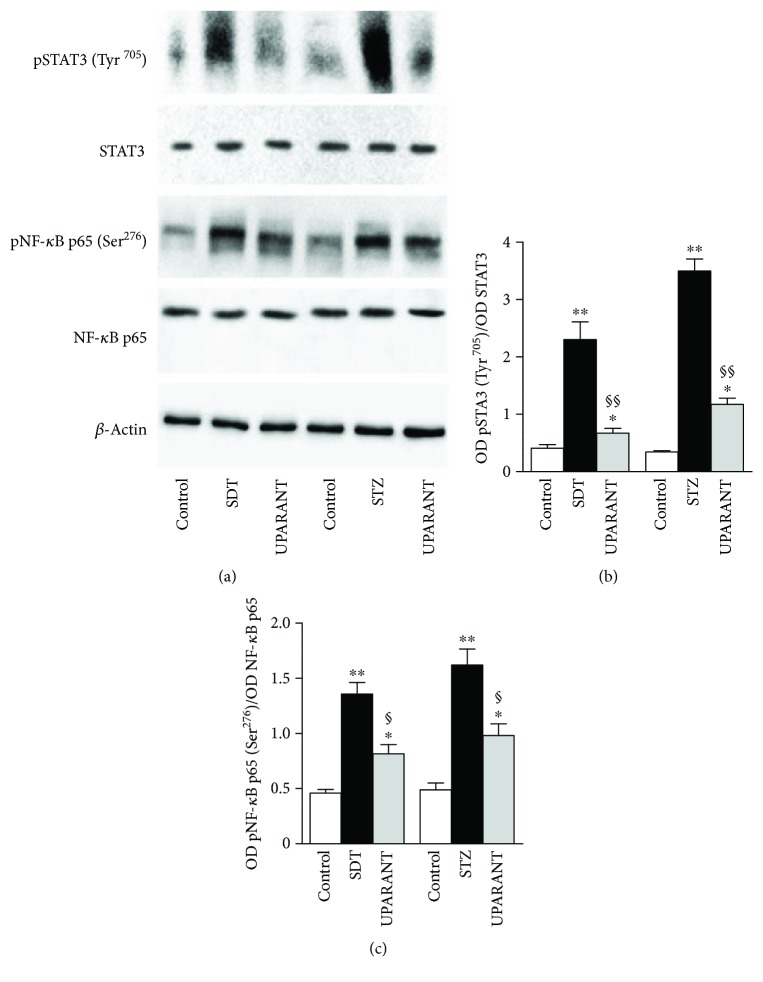
Effects of UPARANT on upregulated levels of transcription factors. (a) Representative blots from SD controls and untreated or UPARANT-treated SDT and STZ rats. (b, c) Protein levels of pSTAT3 (Tyr^205^) (b) and pNF-*κ*B p65 (Ser^276^) (c) were evaluated by the densitometric analysis of the blots depicted in (a) using STAT3 or NF-*κ*B p65 as the loading controls. Hyperglycemia enhanced the phosphorylation of STAT3 at Tyr^705^ and NF-*κ*B p65 at Ser^276^ in both SDT and STZ rats. The increase in transcription factor phosphorylation was significantly reduced by UPARANT in both SDT and STZ rats. ^∗^*P* < 0.01 and ^∗∗^*P* < 0.001 versus control SD rats; ^§^*P* < 0.01 and ^§§^*P* < 0.001 versus untreated SDT or STZ rats (one-way ANOVA followed by Newman–Keuls' multiple comparison posttest; power values: 0.99 (b, c)). Each column represents the mean ± SEM of data from 3 independent samples, each containing 1 retina.

**Figure 12:**
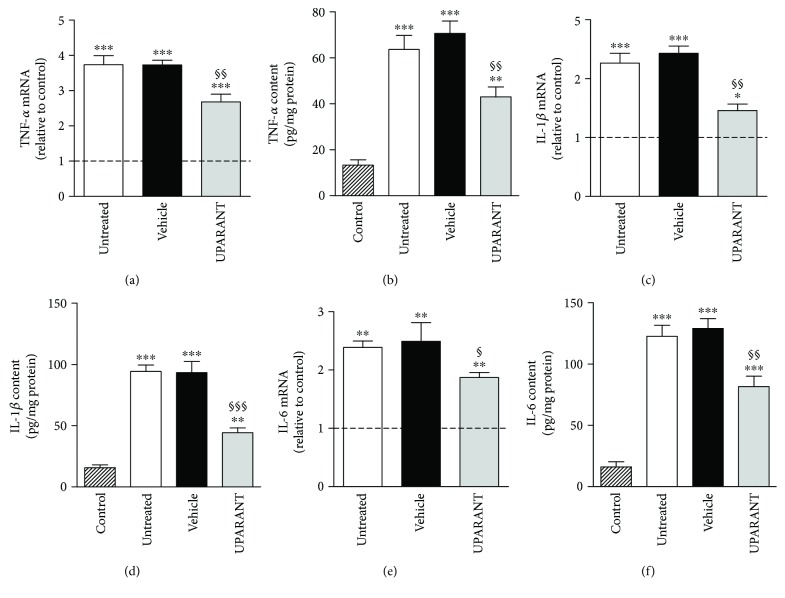
Effects of UPARANT on inflammatory markers. (a, c, e) Transcript levels of TNF-*α* (a), IL-1*β* (c), and GFAP (e) were evaluated by qPCR. Data were analyzed by the formula 2^−ΔΔCT^ using Rpl13a and Hprt as internal standards. (b, d, f) Protein levels of TNF-*α* (b), IL-1*β* (d), and GFAP (f) were evaluated by ELISA. In untreated or vehicle-treated SDT rats, levels of inflammatory markers were increased with respect to SD rats, while UPARANT treatment reduced this increase. ^∗^*P* < 0.05, ^∗∗^*P* < 0.01, and ^∗∗∗^*P* < 0.001 versus control SD rats; ^§^*P* < 0.05, ^§§^*P* < 0.01, and ^§§§^*P* < 0.001 versus vehicle (one-way ANOVA followed by Newman–Keuls' multiple comparison posttest; power values: 0.99 (a–d), 0.85 (e), and 0.99 (f)). Each column represents the mean ± SEM of data from 3 independent samples, each containing 1 retina.

The effects of UPARANT on factors that are downstream to FPRs [16] and are known to mediate the transcription of inflammatory factors, including cytokines [17], were also investigated in both SDT and STZ rats. As shown in Figures 11(a)–11(c), in both models, high glucose increased the phosphorylation of STAT3 at Tyr^705^ and NF-*κ*B p65 at Ser^276^ by about 5.6-fold and 2.9-fold (*P* < 0.001), respectively, in SDT rats, and by about 10.3-fold and 3.3-fold (*P* < 0.001), respectively, in STZ rats. In both SDT and STZ rats, the increase in transcription factor phosphorylation was significantly reduced by UPARANT by 3.4-fold and 1.6-fold in SDT (*P* < 0.001) and by 3.0-fold and 1.7-fold in STZ rats (*P* < 0.01). We also found that untreated SDT rats were characterized by transcript levels of TNF-*α*, IL-1*β*, and IL-6 by 4.0-fold (*P* < 0.001), 2.3-fold (*P* < 0.001), and 2.4-fold (*P* < 0.01) higher than in SD controls (Figures 12(a), 12(c), and 12(e)). A similar increase was also observed in vehicle-treated SDT rats. UPARANT reduced the transcript upregulation by 1.4-fold (*P* < 0.01), 1.7-fold (*P* < 0.01), and 1.3-fold (*P* < 0.05), respectively. Comparable effects of persisting hyperglicemia on inflammatory factors were also determined at the protein level with TNF-*α*, IL-1*β*, and IL-6 in untreated or vehicle-treated SDT rats roughly 5.0-fold, 6.0-fold, and 7.9-fold higher (*P* < 0.001) than in SD controls (Figures 12(b), 12(d), and 12(f)). UPARANT reduced protein upregulation by 1.6-fold (*P* < 0.01), 2.1-fold (*P* < 0.001), and 1.6-fold (*P* < 0.01), respectively.

## 4. Discussion

The present study demonstrates for the first time the preventive efficacy of UPARANT on retinal damage that characterizes the SDT rat as a model approximating type 2 diabetes. Presently, hyperglycemia in type 2 diabetic patients is controlled by oral hypoglycemic and antihyperglycemic drugs although they are not always successful in preventing the onset of DR requiring anti-VEGF treatments that are given when the disease has become vision threatening. A distinctive characteristic of UPARANT is that it prevents DR likely through a dual action, (i) by directly regulating its target expression and (ii) by acting at uPAR crosstalk with FPRs, influencing the activity of transcription factors regulating the expression of inflammatory markers. UPARANT preventive effects demonstrated here also support the effectiveness of systemic administration although its safety in SDT rats still remains to be evaluated.

### 4.1. Characterization of the SDT Model and UPARANT Efficacy

Although no rodent models of type 2 diabetes show the same features of human DR, SDT rats display retinal lesions that closely resemble the human disease including vascular abnormalities leading to reduced barrier properties. Indeed, fluorescein leakage and Evans blue extravasation found here are both indicative of BRB breakdown as also confirmed by altered levels of tight junction components of retinal endothelial cells including claudin-1, claudin-5, and ZO-1 that play an important role in BRB integrity [18]. In particular, they are downregulated in the diabetic retina [19, 20] and in retinal endothelial cells cultured in a high glucose [21, 22]. The additional finding that retinal levels of VEGF and FGF-2 in SDT rats are higher than in SD controls is in line with previous results demonstrating VEGF upregulation in the retina of SDT rats [23, 24]. On the other hand, the lack of alterations in the pattern of retinal vasculature found here is in line with the very low incidence of retinal neovascular formation as described at about 20 weeks after diabetes onset in SDT rats [24]. This is in line with the fact that VEGF not only stimulates vessel proliferation but also plays a role as vasopermeability factor in the diabetic retina [25]. In this respect, Müller cells and activated microglia secrete vasoactive and inflammatory molecules including VEGF [26]. In particular, Müller cells are stimulated by FGF-2 to produce VEGF [27] that, in turn, participates in BRB breakdown by reducing the level of tight junction proteins [28].

Vascular abnormalities leading to reduced barrier properties may be reflected in depressed ERGs, which are established at relatively early stages of the disease [5]. In fact, about 20 weeks of hyperglycemia lead to a 50% reduction in both the a-wave and the b-wave suggesting an involvement of both the outer and the inner retina. On the other hand, hyperglicemia-induced ERG dysfunction is reported even before BRB breakdown indicating that neurodegenerative processes are occurring before the capillary alterations [29]. In this line, additional retinal damages found here including gliosis and retinal cell death are in agreement with previous findings in SDT rats in which GFAP accumulation and caspase-3 activation have been demonstrated in inner and outer retinal layers [30, 31]. In this respect, in the diabetic retina, neurodegenerative processes affect mainly ganglion cells and amacrine cells although photoreceptor death and alterations in the expression of phototrasduction proteins have been also reported [29].

UPARANT dose used here is in line with that used in the STZ model by Cammalleri et al. [12] who also demonstrated that UPARANT reaches the retinal target when subcutaneously administered without any histopathologic alteration of the liver and kidney, the most important organs for detoxification processes, thus indicating the subcutaneous delivery as a promising route to enter the posterior chamber of the eye in the STZ model.

UPARANT-induced prevention of BRB loss, Müller cell gliosis, and retinal cell death shown here in SDT rats is likely to contribute to maintaining retinal integrity as also demonstrated by preventive efficacy of the drug on ERG dysfunction. Preventive action of UPARANT on BRB breakdown has been previously demonstrated in mouse models of neovascular ocular diseases [10, 11]. In addition, UPARANT prevents the VEGF-induced permeability in a monolayer of human retinal endothelial cells [32] and efficiently treats BRB leakage in the STZ model in which VEGF and FGF-2 upregulation is also prevented [12]. In this respect, drugs that reduce retinal levels of VEGF are found to prevent ERG dysfunction or reduce BRB leakage thus limiting DR progression in SDT rats [33–35]. In addition, ranirestat, an inhibitor of the enzyme aldose reductase, which has an early role in the development of DR [36], exerts neuroprotective effects by reducing GFAP accumulation and preventing hyperglycemia-associated structural damage of the retina [31].

### 4.2. Mechanisms Underlying UPARANT Effects

As shown by the present results, the SDT model is characterized by an increased expression of uPAR and FPRs at the transcript and the protein level thus confirming a direct link between their upregulation and DR development. Of the FPRs, FPR2 plays a proinflammatory role as demonstrated in human carotid atherosclerotic lesions, whereas a lower inflammatory response has been observed in macrophages with FPR2 deletion [37]. The additional fact that UPARANT reduces upregulated levels of uPAR, FPR1, and FPR2, without any effect on FPR3, is in line with the finding that the drug has been designed to mimic the sequence through which uPAR interacts with FPR1 and FPR2, but not with FPR3 [9].

Many drug targets thought to be suitable for therapeutic purposes are subjected to positive or negative feedback loops upon chemical perturbations, which might even account for the development of drug tolerance [38]. In particular, members of the G-protein-coupled receptor family when serving as known targets, are regulated upon drug treatment by several mechanisms including receptor desensitization, endocytosis, or regulation of the cellular receptor content [39, 40]. Here, we found that UPARANT treatment in two different rat models of DR, the SDT rat and the STZ rat, achieves the same efficacy, either preventive or curative, through partially different responses. In fact, in the SDT model, UPARANT triggers a negative feedback loop that downregulates the levels of its targets thus presumably rendering the treatment more effective than if it would act at the receptor downstream level only. In the STZ model, in contrast, the uPAR pathway is not influenced by UPARANT suggesting that drug efficacy is solely dependent on potentially switching off the intracellular pathway downstream FPRs. Similarly, in a mouse model of wet AMD, UPARANT has been shown to mitigate laser-induced choroidal neovascularization by inhibiting FPR-mediated regulation of transcription factors coupled to angiogenesis and inflammation without affecting the expression levels of the uPAR/FPR pathway [10].

There are several examples of long-term effects of drugs that are mainly due to the modulation of drug target expression presumably because of the chronic treatment in respect to acute administration. For instance, in rodent models of stroke, the antidiabetic drug metformin can reduce the ischemic events by direcly influencing its molecular targets when administered chronically, but not when administered acutely [41]. In the SDT model, DR lasts several months thus requiring long-lasting treatment, whereas, in the STZ model, UPARANT efficiently counteracts DR signs after 5 days of administration [12]. In both models, UPARANT inhibits FPR-mediated regulation of transcription factors coupled to inflammation. In SDT rats, consequently, preventive administration of UPARANT reduces upregulated levels of inflammatory markers in response to high glucose in line with what found in the STZ model, in which UPARANT decreases inflammation when administered in a therapeutic regimen [12]. Inflammatory factors determined here include inflammatory cytokines such as TNF-*α*, IL-1*β*, and IL6. Among them, TNF-*α* regulates the expression of IL-1*β* and IL-6, while IL-1*β* activates IL-6 production [42]. Inflammatory molecules may induce Müller cell gliosis, as evidenced by increased GFAP expression, and gliotic Müller cells express a wide variety of inflammatory factors, including cytokines [43]. In addition, UPARANT anti-inflammatory activity participates to the inhibition of the angiogenic phenotype by endothelial cells in response to the vitreous fluid from patients with proliferative DR, which is characterized by high levels of angiogenic and inflammatory factors [44]. Moreover, UPARANT action as an anti-inflammatory drug has been recently demonstrated in animal models of inflammation [45]. In this respect, as therapies targeting VEGF do not intervene on inflammatory processes, UPARANT benefits in DR may be greater than those of most anti-VEGF therapies. Consistently, a main role of anti-inflammatory drugs in slowing down the progression of DR lesions has been recently recognized [46].

## 5. Conclusions

The extrapolation of these experimental findings to the clinic is not straightforward although the SDT model approximates at least in part type 2 diabetes. However, the present study provides evidence that preventive administration of UPARANT and continuing regularly along diabetes progression counteracts DR development thus presumably protecting the retina from further worsening of the pathology. In addition, UPARANT preventive effect demonstrated here supports the effectiveness of the systemic route although drug safety still remains to be evaluated. Therefore, the possibility to use a systemic drug that, by slowing down DR progression, may delay the use of intraocularly delivered anti-VEGF agents may be viewed as an added value of UPARANT.

## Figures and Tables

**Table 1:** Sequences of primer sets used for qPCR experiments.

Gene	Primer sequence (5′ → 3′)	
	Forward primer	Reverse primer
*VEGF*	TGTGAGCCTTGTTCAGAGCGG	ACTCAAGCTGCCTCGCCTTGC
*FGF-2*	GCGGCTCTACTGCAAGA	CGTCCATCTTCCTTCATAGC
*Claudin-1*	GTTTCATCCTGGCTTCGCTG	CTTTGCGAAACGCAGGACAT
*Claudin-5*	TACTCAGCACCAAGGCGAAC	TTCCCACATCGGTCTTTCCG
*ZO-1*	AGTCTCGGAAAAGTGCCAGG	GGGCACCATACCAACCATCA
*GFAP*	TGACGCCTCCACTCCCTGCC	CATCTCCGCACGCTCGCTGG
*Caspase-3*	CCTTTCCTCTCCACCGTAGA	AGATGCCACCTCTCCTTTCC
*uPAR*	TTGGATGTTCCTACGAAGAGACG	GTAACTCCGGTTTCCCAGCA
*FPR1*	GTTTCCGCATGAAACGCACT	CATGACCAGGCTGACGATGT
*FPR2*	GCTTCACAATGCCCATGTCC	ACTCGTAAGGGACGACTGGA
*FPR3*	TCCCTTTCAACTGGTTGCCC	GCCAATGAGTTGGTTGGCATA
*TNF-α*	CCCTCACACTCAGTCATCTTCT	GTCACGACGTGGGCTACAG
*IL-1β*	CACCTCTCAAGCAGAGCACAG	GGGTTCCATGGTGAAGTCAAC
*IL-6*	TCCTACCCCAACTTCCAATGCTC	TTGGATGGTCTTGGTCCTTAGCC
*Rpl13a*	GGATCCCTCCACCCTATGACA	CTGGTACTTCCACCCGACCTC
*Hprt*	CTCATGGACTGATTATGGACAGGAC	GCAGGTCAGCAAAGAACTTATAGCC

**Table 2:** Parameters obtained from b-wave amplitude using the Naka-Rushton function.

	SD rats	Untreated SDT rats	Vehicle-treated SDT rats	UPARANT-treated SDT rats
*V*max (*μ*V)	774.80 ± 20.80	456.20 ± 21.62^∗^	500.00 ± 21.96^∗^	758.20 ± 19.83
*k* (log cd-s/m^2^)	−1.47 ± 0.15	−0.84 ± 0.22^∗^	−0.74 ± 0.18^∗^	−1.31 ± 0.14

^∗^ *p* < 0.001 versus SD rats (one-way ANOVA followed by Newman–Keuls' multiple comparison posttest).
